# Supplementary material for: Experience of Service Questionnaire (ESQ) in children and adolescents: factor structure, reliability, validity, item parameters and interpretability of the parent version for practical use in Greece
Source: medRxiv. 2024 Jul 5:2024.07.05.24309986. Preprint. [Version 1] doi: 10.1101/2024.07.05.24309986 (PMC11245072; doi:10.1101/2024.07.05.24309986)
Supplement: Supplement 1 [file media-1.pdf]

Supplementary Material

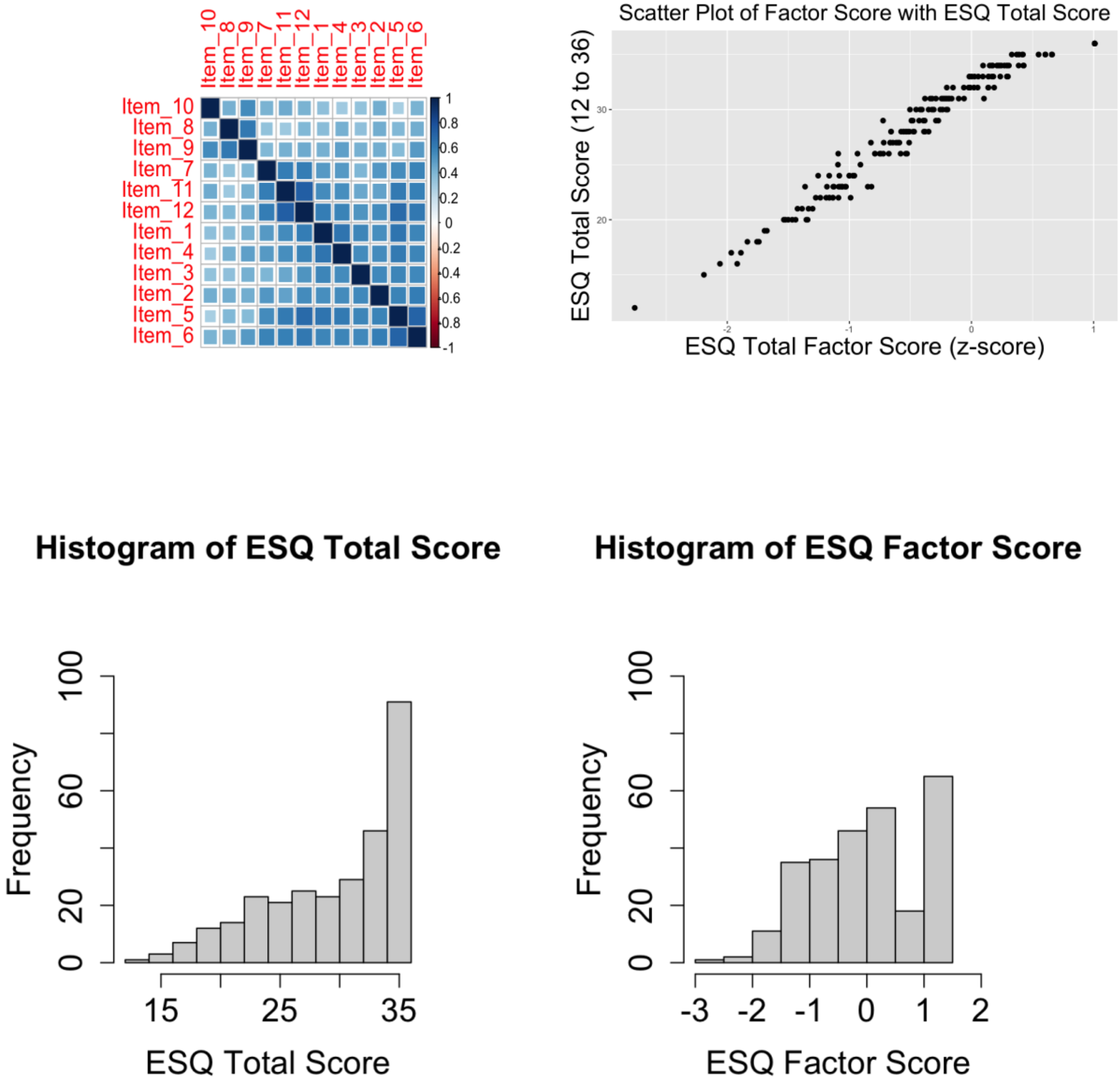

Supplemental Figure S1. Correlation matrix, histograms of the summed-based score and IRT-based score and scatter plot showing the association between summed score and IRT-based score

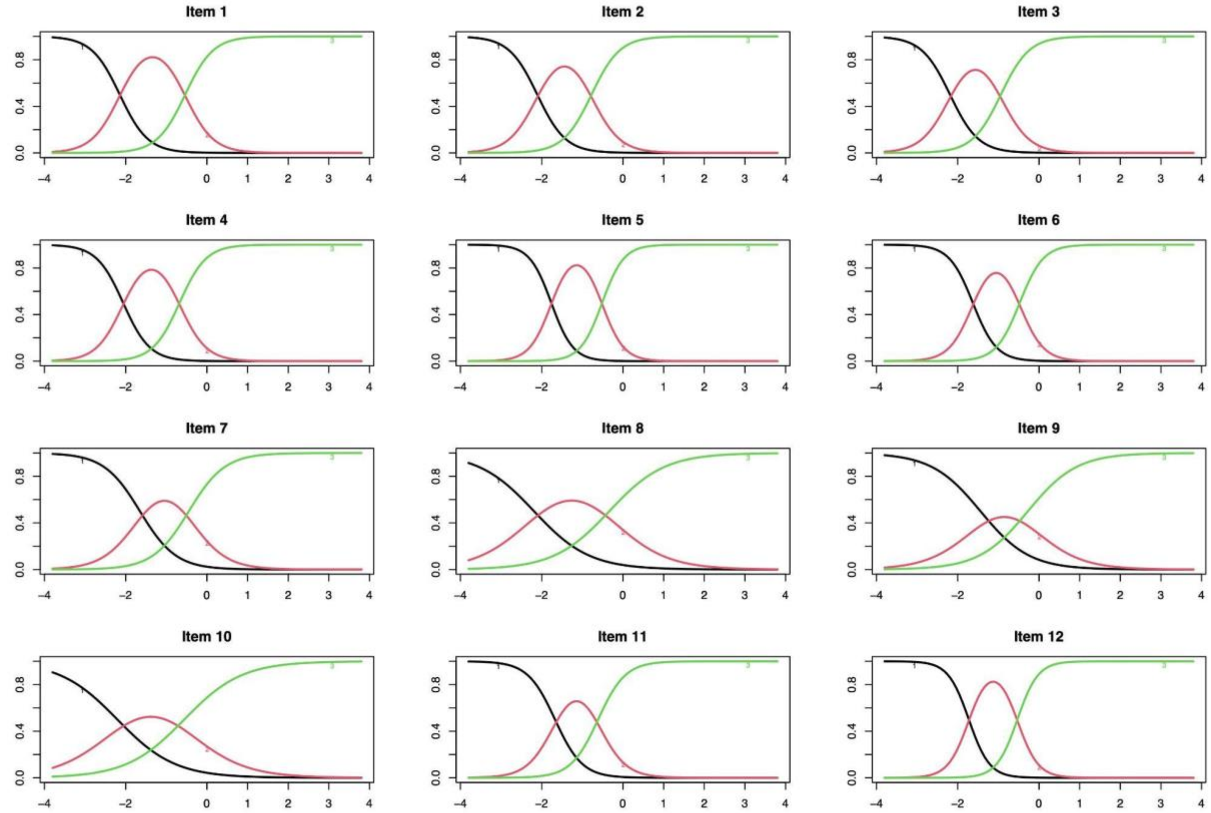

Supplemental Figure S2: Item Response Characteristic Curves (unidimensional solution)

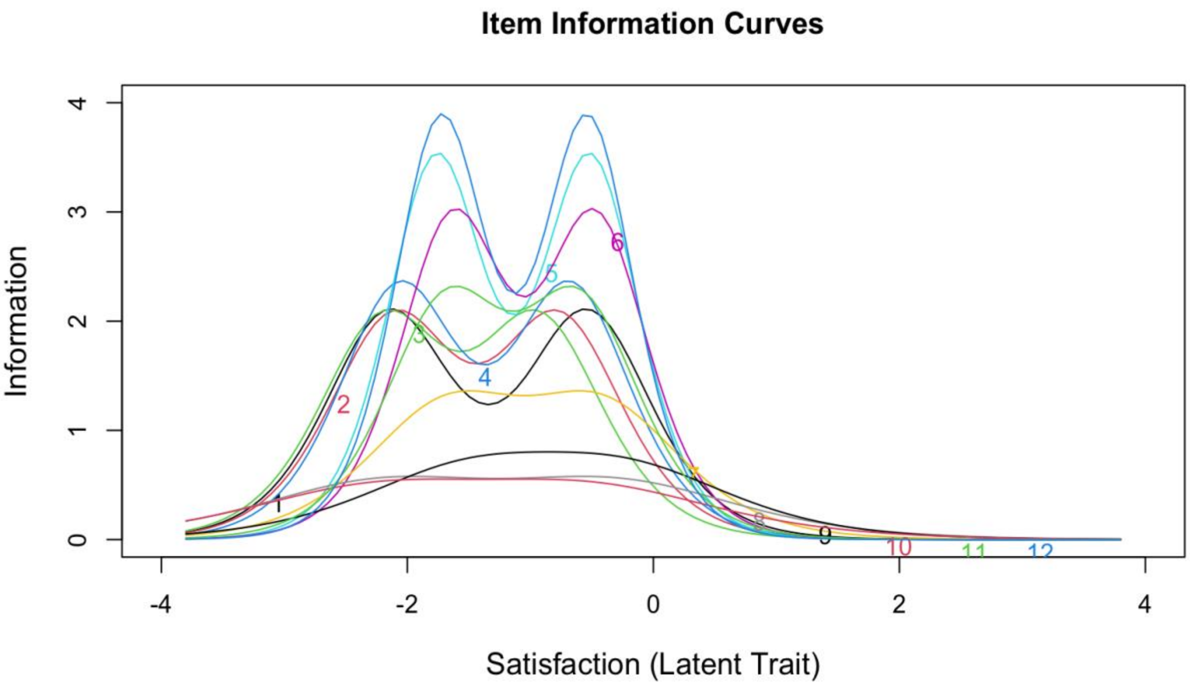

Supplemental Figure S3. Item Information Curves (unidimensional solution)
